# Predicting the dispersal of SARS-CoV-2 RNA from the wastewater treatment plant to the coast

**DOI:** 10.1016/j.heliyon.2022.e10547

**Published:** 2022-09-07

**Authors:** Peter E. Robins, Neil Dickson, Jessica L. Kevill, Shelagh K. Malham, Andrew C. Singer, Richard S. Quilliam, Davey L. Jones

**Affiliations:** a School of Ocean Sciences, Bangor University, Menai Bridge, Anglesey LL59 5AB, UK; b Centre for Environmental Biotechnology, School of Natural Sciences, Bangor University, Bangor, Gwynedd LL57 2UW, UK; c Food Futures Institute, Murdoch University, 90 South Street, Murdoch, WA 6105, Australia; d UK Centre for Ecology & Hydrology, Wallingford OX10 8BB, UK; e Biological and Environmental Sciences, Faculty of Natural Sciences, University of Stirling, Stirling FK9 4LA, UK

**Keywords:** Public health risk, Sewage discharge, Viral surveillance, Water pollution, Wastewater-based epidemiology

## Abstract

Viral pathogens including SARS-CoV-2 RNA have been detected in wastewater treatment effluent, and untreated sewage overflows, that pose an exposure hazard to humans. We assessed whether SARS-CoV-2 RNA was likely to have been present in detectable quantities in UK rivers and estuaries during the first wave of the Covid-19 pandemic. We simulated realistic viral concentrations parameterised on the Camel and Conwy catchments (UK) and their populations, showing detectable SARS-CoV-2 RNA concentrations for untreated but not for treated loading, but also being contingent on viral decay, hydrology, catchment type/shape, and location. Under mean or low river flow conditions, viral RNA concentrated within the estuaries allowing for viral build-up and caused a lag by up to several weeks between the peak in community infections and the viral peak in the environment. There was an increased hazard posed by SARS-CoV-2 RNA with a *T*_*90*_ decay rate >24 h, as the estuarine build-up effect increased. High discharge events transported the viral RNA downstream and offshore, increasing the exposure risk to coastal bathing waters and shellfisheries – although dilution in this case reduced viral concentrations well below detectable levels. Our results highlight the sensitivity of exposure to viral pathogens downstream of wastewater treatment, across a range of viral loadings and catchment characteristics – with implications to environmental surveillance.

## Highlights

• SARS-CoV-2 RNA from treated sewage unlikely to be detectable in estuaries.
• SARS-CoV-2 RNA from untreated sewage can be detectable in estuaries.
• Peak RNA concentration in estuaries can be delayed from peak community infection.
• RNA concentration is sensitive to viral loading, decay, hydrology, and estuary shape.

## Graphical abstract

**Figure:**
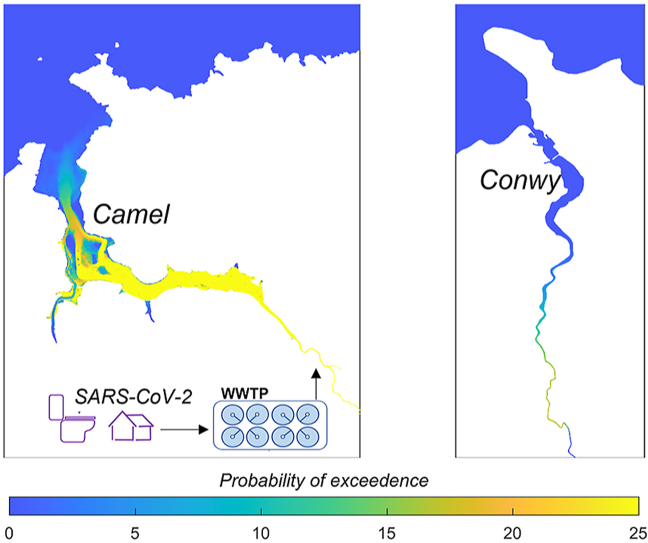

## 1 Introduction

Globally, SARS-CoV-2 RNA has been measured in influent to wastewater treatment plants (WWTPs) to monitor the prevalence of SARS-CoV-2 infections among the community (Ahmed et al., 2020a, 202b; Gonzalez et al., 2020; La Rosa et al., 2020; Medema et al., 2020; Randazzo et al., 2020; Saguti et al., 2021; Sherchan et al., 2020; Westhaus et al., 2021; Gerrity et al., 2021). SARS-CoV-2 RNA has also been detected in wastewater effluent, albeit at lower concentrations, which is subsequently discharged into rivers, estuaries, and coastal waters (Hillary et al., 2021). Raw untreated sewage may also be discharged directly to rivers, especially during high flow and storm events, an occurrence that is not uncommon in the United Kingdom (Hammond et al., 2021). For example, in the UK raw sewage was discharged directly into rivers on 200,000 separate occasions in 2019 (Carver, 2021). This release of microbial pathogens in effluent could pose a theoretical risk of spillover into wildlife vectors (Mathavarajah et al., 2021a, 2021b) and be an infection risk to humans who come in contact with receiving waters (Jones et al., 2020). The infectious nature of SARS-CoV-2 in human faeces and wastewater, however, remains controversial (Pedersen et al., 2021), and may change as new variants of the virus evolve. Sampling of aquatic bodies for SARS-CoV-2 RNA therefore has the potential to contribute to an understanding of the level of infections upstream as well as evaluating the potential infection risk from the water body itself. In regions where wastewater treatment is limited and open defecation is prevalent, direct sampling from rivers and estuaries can still provide information on community-level carriage of infection (Street et al., 2020).

Estimating community levels of infection from the influent wastewater at WWTPs is challenging (Ahmed et al., 2020a, 202b) and involves several assumptions. For example, the following questions need to be considered: what proportion of people infected with Covid-19 have a gastroenteric infection?; how many RNA copies does the average infected person’s faeces contain?; how do faecal shedding rates evolve over the course of an infection?; to what extent is RNA diluted by water in the wastewater system?; what is the time lag in the sewerage network between a defecation event and its arrival at the WWTP?; what proportion of the RNA has decayed before it is assayed? Increasingly, wastewater-derived SARS-CoV-2 data is being relied upon to monitor trends in community infection (Wade et al., 2022); however, there are few examples of sampling from rivers and coastal waters that receive wastewater in order to understand community infection levels (see Street et al., 2020).

Collecting samples from rivers and coastal waters adds an additional challenge, as analyte concentrations can be mediated by hydrological processes and diluted by significant quantities of fresh and/or saline water, potentially negatively impacting the recovery of the analyte. Residence times for contaminated water in estuaries can range from days to months; additionally, estuarine circulation patterns can congregate viruses at certain hotspots (e.g., Brown et al., 1991; Robins et al., 2012). In contrast, high river flows are expected to both dilute and flush viral contaminants offshore depending on the size and geomorphic shape of the estuary (Robins et al., 2018). The effect of these processes on RNA concentrations needs be taken into consideration when working backwards/upstream to infer levels of community infection. To address this challenge, it is essential to develop transferable methods for modelling the transport and persistence of viruses in rivers and estuaries.

There are no known cases of Covid-19 being contracted from wastewater and only a few studies have suggested SARS-CoV-2 can remain infectious in stool (Zhang et al., 2020). It therefore seems unlikely that SARS-CoV-2 RNA present in rivers, estuaries, and coasts poses a real risk to human health. We use SARS-CoV-2 as a tracer for informing future hazard and risk assessments aimed to be maximally protective of recreational water users from microbiological risks originating from treated and untreated wastewater discharged to the aquatic environment.

It is well established in the literature that other viruses and bacteria are much more persistent in wastewater and after being discharged to rivers and estuaries (Pandey et al., 2014). Indeed, thinking beyond the Covid-19 pandemic, developing a better understanding of the spatiotemporal dynamics of microorganisms discharged into rivers and estuaries through wastewater is a pressing research priority. Whilst the modelling presented herein focuses on the first wave of Covid-19 during 2020 in UK river catchments, it also contributes to this broader research priority by investigating the sensitivity of model outputs to a range of parameters (i.e., tracer concentrations, decay rates, treatment factors, river discharge and tide) for two contrasting estuary types.

The two key aims of this study were to: (1) assess the persistence of SARS-CoV-2 RNA; and (2) assess the dynamics of SARS-CoV-2 RNA levels, during the first wave of the Covid-19 pandemic, within two UK rivers and estuaries with different wastewater influent and effluent concentrations, level of wastewater treatment, viral decay rates, catchment hydrology, tidal dynamics and estuary shape.

## 2 Methods

### 2.1 Choice of study catchments

**Figure 1:**
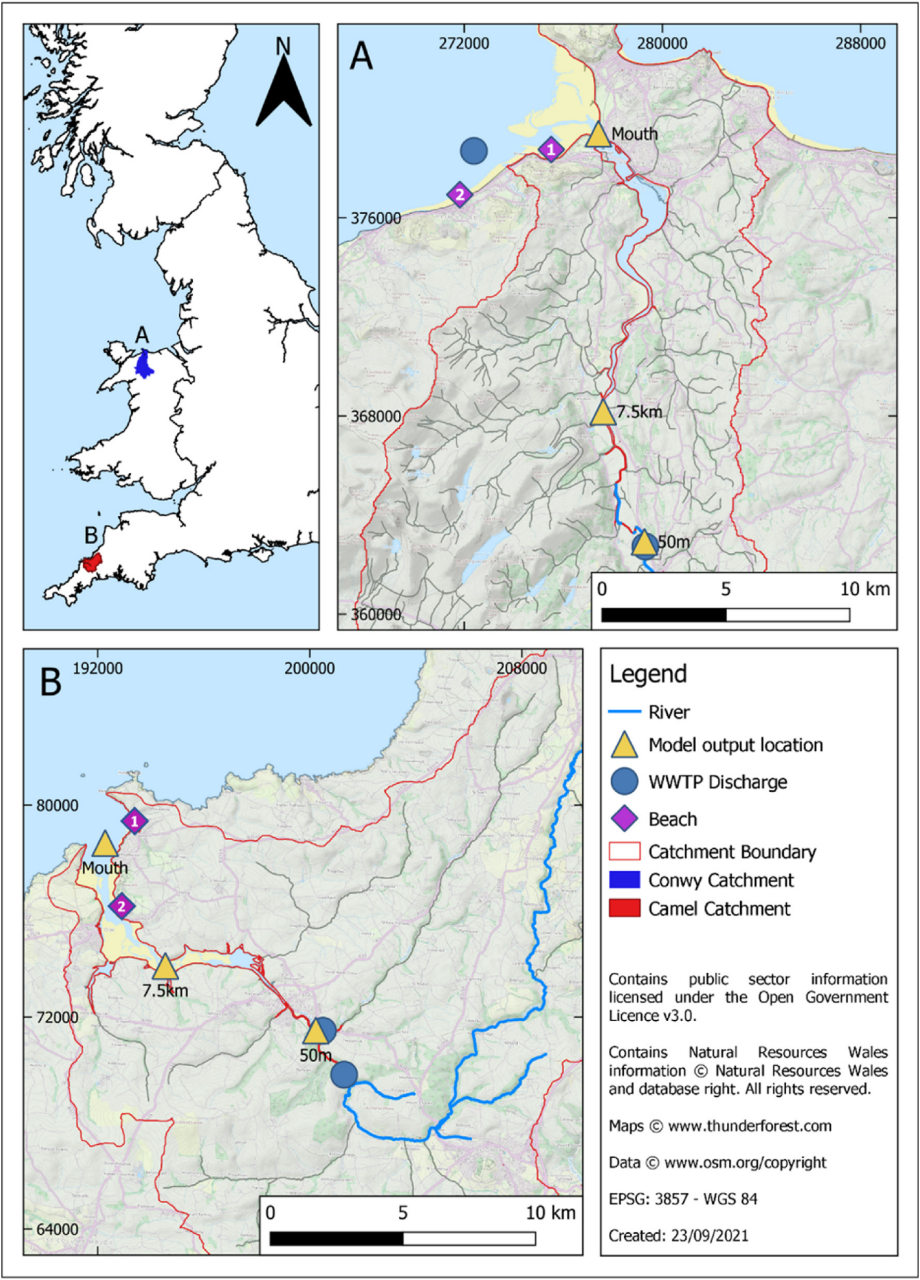
Topography maps of the (A) Conwy and (B) Camel catchments and estuaries (UK). Rivers, WWTP discharges, and popular bathing locations are marked, as well as model output locations used in the analyses.

The Camel (Southwest England) and Conwy (North Wales) catchments (Figure 1 and Table 1) were selected as case studies since extensive data was available for model parametrisation and validation (see Section 2.4), and the hydrodynamics of both estuaries are relatively well studied with reliable river discharge data available from governmental (Environment Agency and Natural Resources Wales) monitoring stations. These systems have contrasting physical behaviours, as described below, which has enabled the role of catchment hydrology and coastal hydrodynamics on viral transport to be studied with greater perspective than could have been gained from studying a single catchment type. Both estuaries are an obvious priority for studying viral transport from wastewater as they contain several active commercial shellfisheries, are flanked with popular bathing and water-sports beaches, and have a known history of wastewater-derived human viral pollution (Adriaenssens et al., 2021).

**Table 1:** Comparison of the Rivers Camel and Conwy catchment characteristics, data summarised from Coxon et al. (2020). Historical data covers the period 1st October 1970 – 30th September 2015. Camel catchment data pertains to the 49001 – Camel at Denby gauge (NERC, 2020b) situated 1.89 km up stream of the upstream model boundary (it does not include the catchments/discharges of the rivers Allen, Polmorfa, Amble and Petherick that were also modelled). Conwy catchment data pertains to the 66011 – Conwy at Cwmlanerch gauge (NERC, 2020b) situated 5.65 km upstream of the upstream model boundary. ∗Negative seasonality index implies precipitation peaks in winter and values close to zero indicate uniform precipitation through the year.

Characteristic	*Camel*	*Conwy*
*Geography and Topology*		
Entire Area Catchment Area (km^2^)	413	574
Upper catchment area - above upstream model limit (km^2^)	210	340
Max Elevation (m)	411	1050
Median Elevation (m)	162	330
Catchment mean drainage path slope	87.9	169.7
*Climatic (historical)*		
Mean daily precipitation (mmd^−1^)	3.77	5.83
Frequency of dry (<1 mm d^−1^) days (d yr^−1^)	188	162
Average duration of dry periods (d)	3.55	3.17
Freq of high-precipitation (≥ 5 times mean daily precipitation) days (d yr^−1^)	14.02	12.53
Average duration of high-precipitation days periods (d)	1.09	1.13
Seasonality Index of precipitation∗	-0.21	-0.26
*Hydrologic (historical)*		
Mean daily discharge (m^3^s^−1^)	2.44	4.82
Q_5_ – 5% flow percentile (m^3^s^−1^)	0.40	0.34
Q_95_ – 95% flow percentile (m^3^s^−1^)	7.11	17.65
Runoff ratio of mean daily discharge to mean daily precipitation	0.65	0.83
Slope of flow duration curves between log transformed 33rd and 66th streamflow percentiles; higher values are generally associated with basins with steeper topography and lower permeability (Yadav et al., 2007)	2.66	3.37
Ratio of mean daily baseflow to daily discharge (based on Ladson et al., 2013 hydrograph separation method)	0.64	0.41
Number of high flow days per year (>9 times the median daily flow)	1.22	13.49

### 2.2 Catchment geography, topology, climate and hydrology

Geographic, topologic, climatic and hydrologic characteristics for the Camel and Conwy catchments, that are fundamental to viral transport, are compared in Table 1. The River Camel flows approximately 48 km from its source on the edge of Bodmin Moor to the north coast of Cornwall (Figure 1) where it discharges from a sandy shallow (intertidal) estuary that only deepens (∼10 m) at its mouth (Environment Agency, 2005). The Camel catchment area is approximately 413 km^2^ with the majority being rural land and only a small proportion urbanised. The rural land is mostly a mixture of pastures and non-irrigated arable land in approximately equal proportions (CEFAS, 2015). Annual mean precipitation varies from 900 mm at the coast to 1800 mm on Bodmin Moor (https://nrfa.ceh.ac.uk/data/station/info/49001).

The River Conwy, on the North Wales coast flows out of the Snowdonia mountain range for around 43 km from source to the open and shallow mouth of Conwy Bay. The catchment is approximately 574 km^2^ with land use predominately a mix of pastoral hill farming, forestry, and mountainous bog/heath. Annual mean precipitation in the catchment increases from 500 mm near the mouth to 3500 mm in parts of the upper catchment (https://nrfa.ceh.ac.uk/data/station/info/66011) – overall precipitation being greater than in the Camel catchment which, in addition to its larger drainage basin, results in a greater mean discharge (4.82 m^3^/s in Conwy vs. 2.44 m^3^/s in Camel; Coxon et al., 2020).

The upper Conwy catchment is more elevated than the Camel catchment and the mean drainage path slopes more severely; these two topographic factors (and impermeable geology of the Conwy) result in faster hydrological pathways within the Conwy than Camel as well as greater relief rainfall. The hydrologic characteristics compared in Table 1 illustrate that relative to the River Camel the hydrology of River Conwy responds more rapidly to rainfall events (greater slope of flow duration curves and greater run-off ratio) and is more amplified by rainfall (higher ratio of mean daily baseflow to daily discharge, and more high-flow days). Indeed, the 5% flow quartile of the River Conwy is less than the Camel (0.34 vs. 0.40 m^3^/s) yet the 95% flow quartile is much greater (7.11 vs. 17.65 m^3^/s; Coxon et al., 2020).

### 2.3 Estuary characteristics

The Camel estuary is a shallow, vertically mixed, predominantly sandy ria (Oyedotun et al., 2013). It is macrotidal with a mean spring tidal range of 6–7 m at the mouth, decreasing towards the estuary head 12 km upstream. The intertidal area is approximately 6 km^2^, much of which is tidal flats, but also saltmarshes, subtidal channels and dunes (Brew and Gibberd, 2009). The outer estuary is approximately 1 km wide but narrows just north of Padstow: landward of this neck, the inner estuary is more sheltered, which enables the estuary to function as an important sink for particulates (Brew and Gibberd, 2009).

The Conwy estuary is a shallow, (largely) vertically mixed, predominantly sandy embayment (Simpson et al., 2001; Howlett et al., 2015). Like the Camel, the Conwy is macrotidal with a similar spring tidal range of 6–7 m but is a longer estuary (20 km) whilst being of a similar intertidal area (6 km^2^) (Robins et al., 2019). The morphology is such that the estuary almost entirely drains each tidal cycle, and resembles a meandering river channel at low water, flanked by narrow mud flats in the upper estuary and sand in the lower estuary, and does not contain extensive saltmarshes (Robins et al., 2014). Similar to the Camel, the Conwy estuary also narrows just upstream of the mouth (at the Conwy Bridge crossing), causing a sheltered inner estuary that is potentially a particulate sink. Both the Camel and Conwy estuary experience an axial convergent front, which can further retain particulates within the estuary (Brown et al., 1991).

### 2.4 Population and wastewater treatment

The Camel catchment has a resident population of 59,579. This population is served by 11 secondary (chemical and biological) and six tertiary (UV) wastewater treatment plants (WWTPs) providing different levels of treatment to wastewater. Three of the treatment plants discharge directly into the saline estuary whilst the remaining 14 discharge into the River Camel or one of its tributaries upstream of the saline intrusion.

The Conwy catchment, including the coastal settlements of Llandudno, Colwyn Bay, Deganwy and Llandudno Junction, has a resident population of around 78,600. Much of the coastal population’s wastewater receives UV treatment before being pumped 1.6 km offshore to the Ganol STW outfall (i.e., offshore of the estuary mouth). A population of 11,506 (Dŵr Cymru Welsh Water, pers. comm.) is served by 24 WWTP’s that discharge directly into the River Conwy/Conwy Estuary which predominantly receives secondary-level treatment (Biological Filtration) except for two small WWTPs that use a septic tank or package treatment. Ten of the 24 WWTPs discharge downstream of the tidal limit (Llanrwst) and 14 discharge further upstream. There is an additional WWTP off-shore of Penmaenmawr that was included in this study as its near-by location in Conwy Bay makes it a potential source of contamination to shellfish beds and bathing waters around the estuary mouth.

In addition to the continuous wastewater discharges from WWTP’s there are a further 58 water company owned Combined Sewer Overflows (CSOs) in the Camel catchment and 54 in the Conwy. These CSOs tend to operate following high rainfall events but also at other times throughout the year (CEFAS, 2014, 2015). Reliable spill data, inclusive of volume of discharge, was not available at these sites (or any other CSO in the UK), as such, the effect of CSO discharges was not considered in this study.

### 2.5 Modelling virus load to the estuary from the catchment

**Figure 2:**
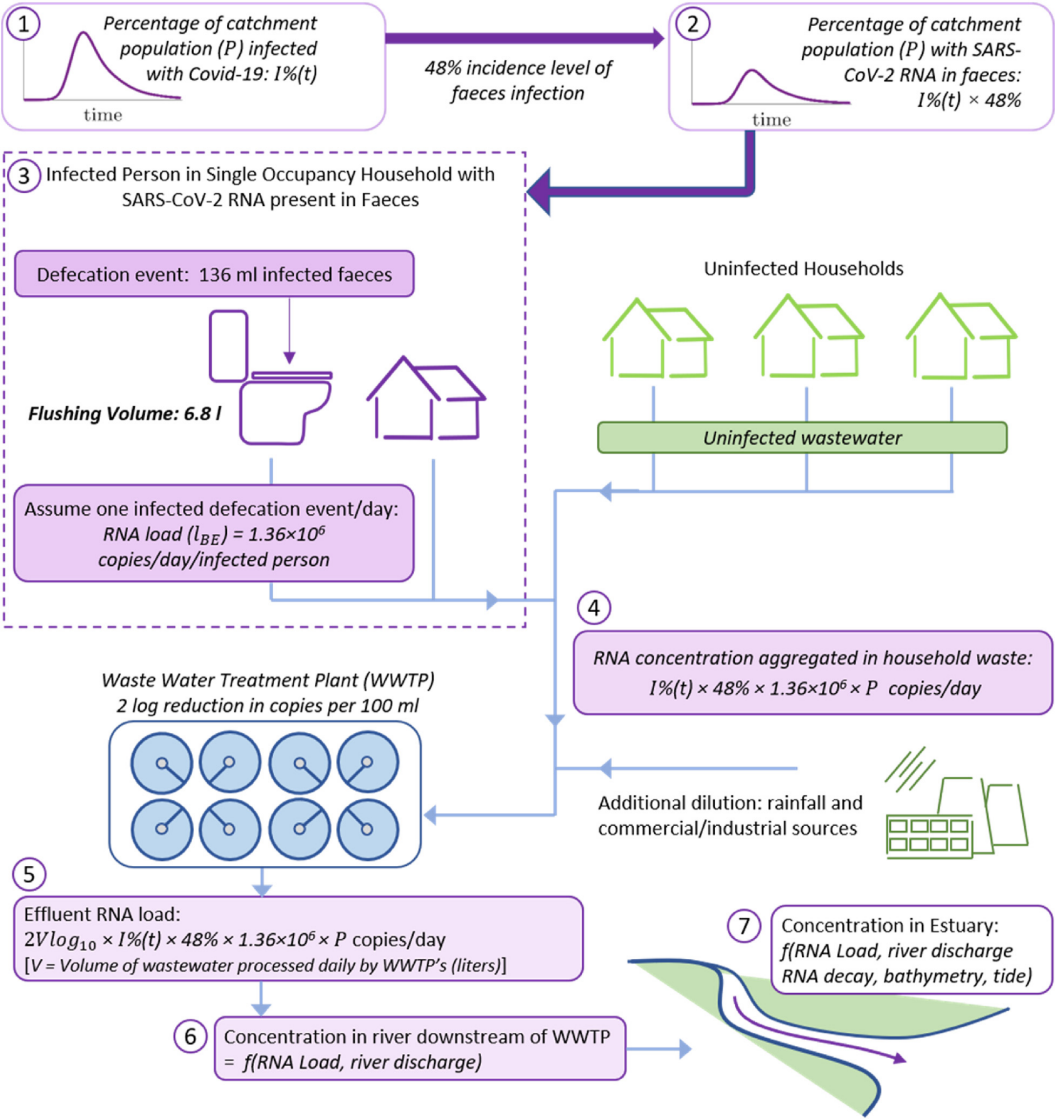
Conceptual dilution/transport model of SARS-CoV-2 RNA copies from person to estuary.

The framework that was used to model the transport and dilution of SARS-CoV-2 RNA is summarised in Figure 2. To model the transport of the RNA within rivers and estuaries it was first necessary to approximate the loading to the rivers/estuaries from the wastewater sites. The RNA load to the estuaries was calculated based on estimates of the number of prevalent infections within the catchments, an estimate of the proportion of infected people with RNA present in their faeces, the load contributed per infected person, and an assumed reduction in viral load through wastewater treatment. This load was then divided and discharged at specific inputs within the model domain according to the WWTP locations. Further details follow on the assumptions made to arrive at the WWTP RNA effluent loads and the different scenarios that were modelled (summarised in Table 2).

**Table 2:** *Modelled scenarios of* SARS-CoV-2 RNA loading in the Camel and Conwy estuaries during spring-summer 2020. Note: each scenario was run twice with different decay rates; T_90_ = 24 h and T_90_ =35 ​days (840 h).

Simulation		Infection timeseries I(t)	RNA load of Infected person with positive faeces (copies/day)	2 Log_10_ Treatment	Number of effluent discharge locations (Camel, Conwy)	Catchment Population (Camel; Conwy)
1	*Best Estimate Loading*	*ONS*	$1.36\times 10^{6}$	*Yes*	*3,2*	59,579; *11,506*
*2*	*IMHE infections*	*IMHE*	$1.36\times 10^{6}$	*Yes*	*3,2*	59,579; *11,506*
*3*	*Worse Case Loading*	*ONS*	$6.80\times 10^{6}$	*Yes*	*3,2*	59,579; *11,506.*
*4*	*Best Estimate Untreated*	*ONS*	$1.36\times 10^{6}$	*No*	*3,2*	59,579; *11,506*
*5*	*Worse Case Untreated*	*ONS*	$6.80\times 10^{6}$	*No*	*3,2*	59,579; *11,506*
*6*	*Equal loadings Treated*	*ONS*	$1.36\times 10^{6}$	*Yes*	*1,1*	59,579; 59,579
*7*	*Equal loadings Untreated*	*ONS*	$1.36\times 10^{6}$	*No*	*1,1*	59,579; 59,579

#### 2.5.1 Estimating the number of prevalent infections within the catchments

**Figure 3:**
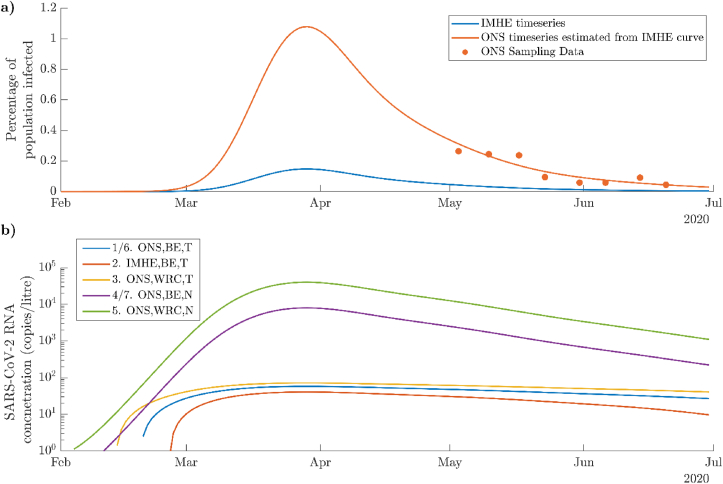
(a) Percentage of population infected curves used, (b) SARS-CoV-2 RNA concentrations for WWTP effluent discharges for simulation. Numbers 1–5 in legend correspond to Simulations 1–5 in Table 2.

Daily estimates of the number of prevalent infections in the UK were obtained from the Institute of Health Metrics and Evaluation (IHME) between 4th February and 28th June 2020 (IMHE, 2020b). The IMHE define prevalent infections ‘as all cases that exist on a location on a given day, not just new ones’ (IMHE, 2020a) and estimate this value by working backwards from death estimates using infection fatality ratios. To provide a ‘spin-up’ modelling period the data was extended back to 1st Jan 2020 with zero daily values of prevalent infections. The data set was extended beyond 17th June through to 28th June 2020 using IMHE estimates made on 29th June 2020 (IMHE 2020a); see Figure 3a.

A second timeseries of daily estimates of infections for the same period was synthesised based on data from the Office for National Statistics (ONS) Coronavirus Infection Surveys (ONS, 2020a) for infections in England (only), which recorded eight surveys between 27th April and 27th June 2020. This data was extrapolated backwards in time through the first wave of the pandemic using a scale factor based on the IMHE results from the period 27th April to 27th June, and then interpolated to a daily frequency. Firstly, IMHE and ONS data sets were re-scaled as a percentage of the population; populations of England and UK taken as 56.98 M and 66.80 M, respectively (ONS, 2020b). IMHE estimates were then noted on the median date of each of the eight ONS surveys; for each of these dates the percentage of infections according to the ONS survey $\left(I^{ONS}\right)$ were divided by the percentage of infections according to the IMHE timeseries $\left(I^{IMHE}\right)$. The mean of these eight values was then calculated (Eq.(1)) to obtain a scale factor which was used to scale the IMHE prevalent infection timeseries between 4th Feb and 28th June 2020 (Figure 3a):

(1) $$Scale\;factor=\frac{1}{8}\overset{8}{\underset{i=1}{\sum }}\frac{I_{i}^{ONS}}{I_{i}^{IMHE}}$$

#### 2.5.2 Estimating viral load contributions per infected person

A number of studies have detected SARS-CoV-2 RNA in the faeces of hospitalised patients infected with SARS-CoV-2; in cases with and without gastrointestinal symptoms or diarrhoea (e.g. Chen et al., 2020; Lin et al., 2020; Wang et al., 2020; Wu et al., 2022). However, studies have indicated that faeces positivity only occurs in a proportion of SARS-CoV-2 patients (up to 75%) that have been diagnosed via a respiratory test (Cheung et al., 2020; Mesoraca et al., 2020; Zheng et al., 2020; Zhang et al., 2021; Moura et al., 2022). Whereas model studies of community-wide SARS-CoV-2 RNA have assumed faecal shedding in all cases, but with varying loads, to match wastewater surveillance samples (Hoffmann and Alsing, 2021; Wu et al., 2022). We used the assumption that 50% of those infected with SARS-CoV-2 would have the RNA present in their faeces based on the findings of a meta-analysis of 60 studies comprising of 4243 patients (Cheung et al., 2020). SARS-CoV-2 has also been detected in the urine of patients, however, the majority of studies that have tested for urine contamination have reported negative results (Foladori et al., 2020; Jones et al., 2020); cases of detection have been rare (1 in 9 patients: Peng et al., 2020; 1/96 patients: Zheng et al., 2020) and where reported concentrations have been very low (3.22 × 10^2^ copies/ml: Peng et al., 2020). Therefore, we have not included SARS-CoV-2 loading to our model via urine inputs.

The quantity of SARS-CoV-2 RNA shedding that occurs in faeces varies significantly between people and temporally over the course of an infection (Jones et al., 2020; Zheng et al., 2020; Moura et al., 2022; Wu et al., 2022). All published studies have focused on hospitalised patients and maximum loads of up to 1.0 × 10^7^ copies/ml have been reported in severely ill patients (Han et al., 2020; Wölfel et al., 2020). Zheng et al. (2020) reported a median SARS-CoV-2 RNA load of 5 $\;\times 10^{4}\;\text{copies}/\text{ml}$ in positive faeces (N = 93; total patients in study), whilst Cheung et al. (2020) found median loading of comparable magnitude 4.1 $\times 10^{4}$ copies/ml only in patients with diarrhoea (N = 59, total patients in study). Cheung et al. (2020) reported lower median loads; 7.9 $\times 10^{3}$ copies/ml amongst patients without diarrhoea. We modelled two different loading scenarios: (1) a ‘best estimate’ (BE) scenario where we assumed the RNA concentration in infected faeces was, $C_{BE}=1\times 10^{4}\;\text{copies}/\text{ml}$; and (2) a ‘worst case’ (WC) scenario, $C_{WC}=5\times 10^{4}\;\text{copies}/\text{ml}$. This WC threshold RNA concentration in infected faeces is similar or higher across other microbial hazards (e.g. human parechoviruses: $10^{4}-$$10^{8}$ copies/ml (Baumgarte et al., 2008); *E. coli*: $10^{6}$ copies/ml (Pang et al., 2004); Norovirus: $10^{5}-10^{9}$ copies/ml (Teunis et al., 2015)).

The volume of faeces excreted by each infected person per day (V) was assumed to be 136ml which was calculated based on an assumed faecal wet mass of 128g/person/day and density 1.06g/ml (Rose et al., 2015). Thus, we calculated two values for the infected load (l) per person with faecal SARS-CoV-2 RNA positivity: $l_{BE}=1.36\times 10^{6}\;\text{copies}/\text{ml}$ and $l_{WC}=6.80\times 10^{4}\;\text{copies}/\text{ml}$, where l=V×C.

The daily viral load (L(t)) of SARS-CoV-2 to wastewater influent by infected persons within each catchment with population (P) was thus calculated by (Eq.(2)):

(2) L(t)=I%×50%×l×Pcopies/day

#### 2.5.3 Effect of wastewater treatment on viral load

WWTPs result in a significant reduction in viral load, with primary treatment reportedly resulting in a $1log_{10}-6log_{10}$ removal in viral load (Bogler et al., 2020; Wurtzer et al., 2020; Saba et al., 2021), although this varies country to country. For our simulations based on UK WWTPs, a reduction in tracer concentration (expressed as cfu/100 ml) of 2 $log_{10}$ was used in simulations 1–3 and 6 (see Table 2; Hillary et al., 2021). To apply this reduction, it was necessary to convert the viral load to a concentration to estimate the volume of treated waste. It was not possible to obtain data on volumes processed in the Conwy and Camel catchments. However, the average daily volume processed by Bangor WWTP was known (1.0 ${\text{m}}^{3}/\text{population}/\text{day}$) and used to scale to the Conwy and Camel catchments, as these are all similar in scale, land use, and population. Based on Bangor WWTP mean discharge ($Q_{Ban}$) expressed in ${\text{m}}^{3}/\text{day ​}$ and the population it serves ($P_{Ban}$) the daily discharge volume of wastewater to the Camel ($Q_{Cam}$) and Conwy $(Q_{Con}$) catchments expressed in ${\text{m}}^{3}/\text{day}$ were estimated by:

(3a) $$Q_{Cam}=Q_{Ban}\times \frac{P_{Cam}}{P_{Ban}}$$

(3b) $$Q_{Con}=Q_{Ban}\times \frac{P_{Con}}{P_{Ban}}$$

The concentration of contaminated wastewater in the Camel ($C_{cam}\left(t\right)$) and Conwy ($C_{Con}\left(t\right)$) catchments expressed in cfu/100ml was thus calculated by:

(4a) $$C_{Cam}\left(t\right)=\frac{1\;}{10,000}\times \frac{L_{Cam}\left(\text{t}\right)}{Q_{Con}}$$

(4b) $$C_{Con}\left(t\right)=\frac{1\;}{10,000}\;\times \frac{L_{Con}\left(\text{t}\right)}{Q_{Con}}$$

These concentrations (Eqs. (4a) and (4b)) were reduced by a $2log_{10}$ transformation to give the post-treatment effluent concentration of SARS-CoV-2 (Hillary et al., 2021). Treated and untreated SARS-CoV-2 RNA concentration curves in each of the simulations detailed in Table 2 are presented in Figure 3b.

#### 2.5.4 Dividing catchment effluent load to give model inputs

The spatial loading of effluent discharge and thus SARS-CoV-2 RNA to the River Camel was derived by distributing $Q_{Cam}$ based on estimated loadings of *E. coli* for the catchment to each of its WWTPs made by CEFAS (2015); these are detailed in Appendix 1. 98.5% of the effluent load was associated with WWTPs discharging into the Rivers Allen, Amble or Camel upstream of the model domain (Figure 2). The upstream model forcing boundaries where each of these rivers enters the model domain were spiked with time dependent virtual tracers representing SARS-CoV-2 RNA proportional to each of the $C_{Cam}\left(t\right)$ or $2log_{10}C_{Cam}\left(t\right)$ timeseries, weighted by the factors given in Table 3. These factors were derived by summing the effluent loading percentages of sites upstream (Appendix 1). Loadings from other WWTPs on other rivers, that contributed 1.5% of the total loadings to the Camel estuary, were incorporated in the existing WWTPs to simplify the modelled system yet retain the overall loading to the system.

**Table 3:** Apportioning of effluent load to locations in the Camel and Conwy model domains.

Ref	Location in Model	Lat, Long	Effluent loading weighting
**Camel Catchment**			
Cam_WS	River Allen Upstream Boundary	50.4928, −4.8030	51.6%
All_WS	River Camel Upstream Boundary	50.5084, −4.8132	47.0%
Amb_WS	River Amble Upstream Boundary	50.5325, −4.8491	1.5%
**Conwy Catchment**			
Con_WS	River Conwy Upstream Boundary	53.1490, −3.8068	58.9%
Pen_WS	Penmaenmawr Coastal Waters	53.2900, −3.9127	41.1%

A similar approach to apportioning effluent load was adopted for the Conwy catchment. As detailed in Table 3, 46.3% of apportioned effluent load was associated with WWTPs discharging into the River Conwy upstream of the model domain, 41.1% with Penmaenmawr WWTP which discharges into the coastal waters of Conwy Bay and the remaining 12.6% from 10 small WWTPs situated between the model upstream river boundary (just upstream of the tidal limit) and the coast. To simplify the system, the load for these 10 WWTPs was aggregated with the load from upstream WWTPs. Therefore, the SARS-CoV-2 RNA loading ($C_{Con}\left(t\right)$ or $2log_{10}C_{Con}\left(t\right)$) was inputted to the model as a virtual tracer from the model upstream river boundaries, at Llanwrst accounting for 58.9% of the catchments effluent load, and at the location of the Penmaenmawr WWTP (Figure 1) accounting for 41.1% of the catchments effluent load.

To focus on comparing the influence of the hydrodynamics of each river/estuary on tracer transport, for the final simulations 6 and 7, both catchments were assumed to have the same population $\left(P_{Cam}=59,579\right)$ and thus loaded with equal tracer loadings. In these simulations a single tracer discharge location was used at the location of the largest WWTP in each model (Figure 1).

#### 2.5.5 Viral decay rates

SARS-CoV-2 decay rates of *T*_*90*_ = 1 day and *T*_*90*_ = 35 days (840 h) were simulated for each of the scenarios presented in Table 2; *T*_*90*_ refers to the time taken for 90% of the viral copies to decay to a level where they are no longer detectable. *T*_*90*_ values of 1 and 35 days were selected as lower and upper limits based upon the current scientific knowledge available. Although our decay parameters are fitted to SARS-CoV-2, there are several other viral pathogens that will have decay rates of several weeks (Dean and Mitchell, 2022). To date, research to establish a *T*_*90*_ involves SARS-CoV-2 seeded wastewater, stored at different temperatures in a controlled setting and as such the *T*_*90*_ values vary between studies (Ahmed et al., 2020a, 202b; Bivins et al., 2020; de Oliveira et al., 2021). At 20 °C and 24 °C the *T*_*90*_ is 2.1 and 1.2 days, respectively (Bivins et al., 2020; de Oliveira et al., 2021); however, a *T*_*90*_ of 12.6 days has been reported for wastewater stored at 25 °C (Ahmed et al., 2020a, 202b). UK sewer temperatures range between 10 °C and 25 °C with a yearly average of 17 °C (Abdel-Aal et al., 2018; Ali and Gillich, 2019). Therefore, the approximate *T*_*90*_ thresholds of 1 day and 35 days were selected to account these seawater temperature ranges. Note that we emphasise this is distinct from the time taken for the virus to decay to a state where it is no longer infectious (i.e., insufficient copies to cause infection or the infectivity of the virus has been compromised due to decay); it seems unlikely that any virus would remain infectious following its passage through the sewerage network and WWTP treatment, however, this cannot be discounted where raw untreated sewerage is discharged directly into rivers/estuaries.

### 2.6 Hydrodynamic model details

We used a hydrodynamic model (Telemac Modelling System V7.2; www.opentelemac.org) to simulate the dispersal of SARS-CoV-2 within the Conwy and Camel estuaries. The Telemac model is well-suited to vertically mixed coastal and estuarine applications due to its unstructured grid configuration that can be optimised to adequately resolve coastal topography and bathymetric features (e.g., <50 m scale) and associated near-coast dynamic circulation and mixing that are important for pollutant dispersal, and the model incorporates wetting and drying capabilities of inter-tidal regions. Telemac is used widely across the global scientific community, with recent relevant applications including Gracia et al. (2018); Yang et al. (2019); and Guo et al. (2020). Telemac is a hydrostatic model that computes the depth-averaged shallow water Saint-Venant equations of momentum and continuity on an unstructured triangular mesh (Hervouet, 2007). The mesh is mapped on to observational bathymetry data from each estuary. The bathymetric data was obtained from several sources: (1) the UK Government’s ADMIRALTY Marine Data Portal (www.admiralty.co.uk/ukho) at 200 m spatial resolution (EDINA, 2008); (2) LIDAR data in coastal/intertidal regions at 10 m resolution (available from the UK Environment Agency and Natural Resources Wales), surveyed in 2011 (Conwy) and 2018 (Camel); (3) multibeam surveys of the coast surrounding the Conwy at 10 m resolution conducted by Bangor University in 2013; and (4) single-beam echosounder surveys of the sub-tidal Conwy estuary channel which was conducted by Bangor University in 2003 and 2019.

Within Telemac, the k-ε turbulence model has been parameterised into vertically averaged form (Rastogi and Rodi, 1978), and Nikuradse’s law of bottom friction was used with a constant friction coefficient of 3 × 10^−2^ m (Hervouet, 2007). A constant turbulent viscosity was set with an overall (molecular + turbulent) viscosity coefficient of 10^−6^.

The two models were spun-up during one month to create a steady-state salinity balance under low river flow conditions (Conwy Q_5_ flow of 0.34 m^3^/s; Camel Q_5_ flow of 0.4 m^3^/s). Tidal forcing at open boundaries (both elevations and velocities) comprised the following harmonic constituents: M_2_, S_2_, N_2_, K_2_, K_1_, O_1_, P_1_, Q_1_, M_4_, MS_4_, MN_4_, M_f_ and M_m_, derived from the TPXO global tidal database of 0.25° resolution (Egbert et al., 1994). Model validation have previously been conducted for elevations, depth-averaged velocities and the salinity structure (see Robins et al., 2014), which test the suitability of the Telemac for application to the Conwy.

Next, scenarios 1–7 (see Table 2) were simulated with both the Conwy and Camel models for the period 01 March to 31 July 2020. River forcing comprised 15-minute discharge data that was applied at the position of the river gauging stations on the main rivers, above the tidal influence. For all simulations, key parameters (depth, velocity, salinity, virus concentration) were output every 15 min.

**Figure 4:**
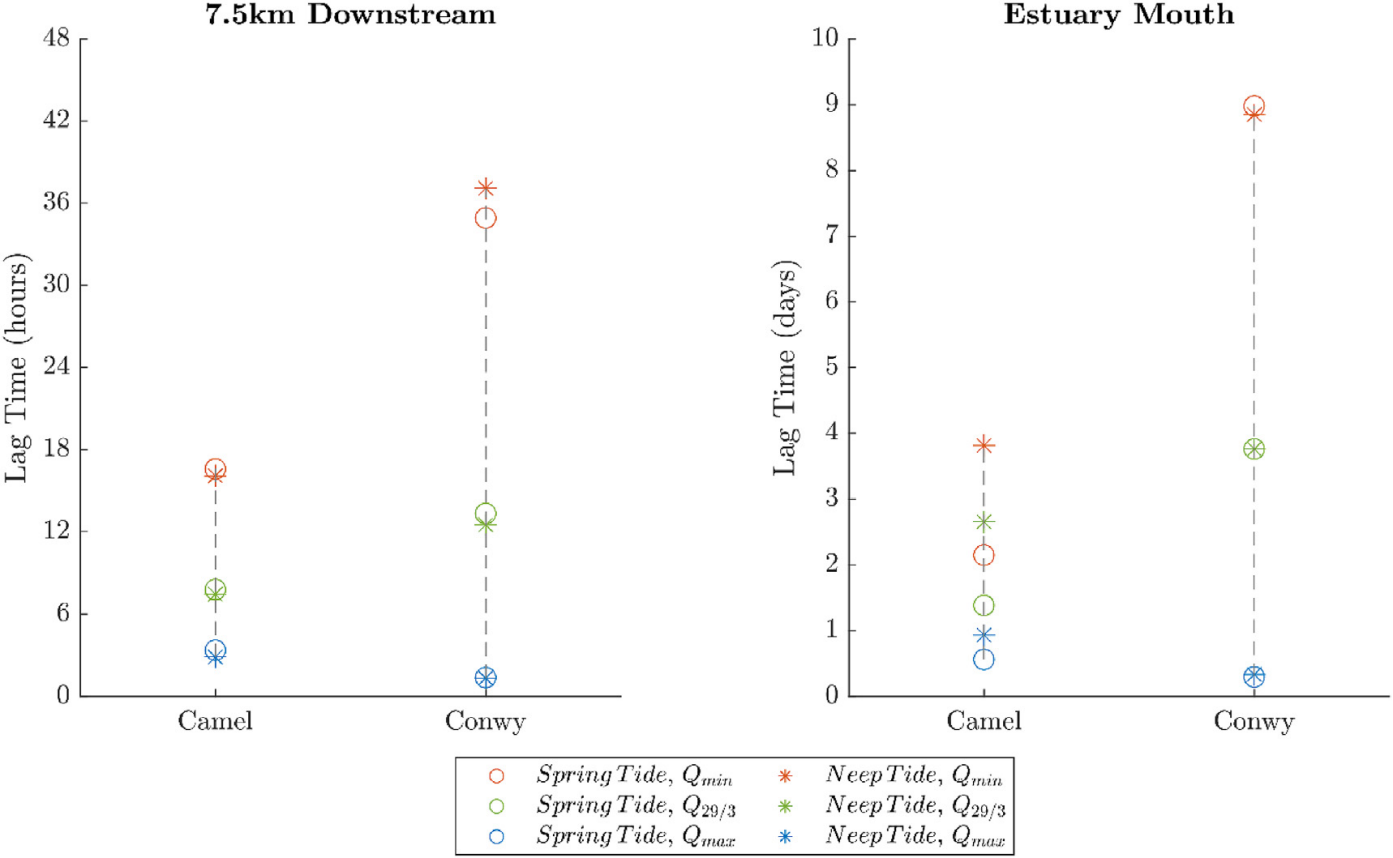
Lag times for the Camel and Conwy estuaries under different flow and tidal conditions based on the release of tracers from the upstream loading points in Simulations 6/7 (see Figure 1) to a point 7.5km downstream (left) and the estuary mouths (right). Note that the distances to the estuary mouths from the upstream loading points (measured along the river thalwegs) are not equal; 13.6 km and 21.0 km for the Camel and Conwy, respectively. Each data point plotted is the mean lag time from four tracer releases at (i) high tide, (ii) mid-ebb, (iii) low tide and (iv) mid-flood.

To assess the transport times of SARS-CoV-2 RNA in each river under different tidal and river flow conditions a separate series of simulations were run where pulses of tracer were discharged at the same locations as the loading points as in Sim 6/7 and the time taken for tracer concentrations to peak at locations downstream recorded (Figure 4). Simulations were carried out with three different river discharges (discharge was constant over each simulation period); (i) Q_min_, the min mean daily discharge recorded over the study period, (ii) Q_29/3,_ the mean daily discharge recorded on the 29th March 2020 which was date of the peak of simulated infections, (iii) Q_max_, the max mean daily discharge recorded over the study period. For each discharge rate, simulations were repeated commencing on the largest spring tide of the study period (11th March) and the smallest neap tide (19th March) giving in total 12 simulations; 6 for each river (further details are given in Table 4). Under each set of river flow/tidal conditions, four uniquely identifiable tracer pulses were released each lasting 1 min: the first at high tide then subsequentially at 3 h 6 min 15 s (thus equally spaced over a diurnal tide cycle).

**Table 4:** Details of river discharge and high tide times used for transport time simulations. The Camel the discharge given is for the main river, the discharge of its four tributaries included in the model were scaled proportionally.

	Camel	Conwy
	***Discharge(m***^***3***^***s***^***−1***^***)***	
*Q*_*min*_	*0.82*	*0.99*
*Q*_*29/3*_	*4.82*	*4.62*
*Q*_*max*_	*35.80*	*312.99*
	***Max tidal elevation above mean (m)***	
*Spring Tide*	*6.00m*	*5.96m*
*Neap Tide*	*1.36m*	*1.80m*

### 2.7 Post simulation analysis

To investigate the potential for detecting SARS-CoV-2 in the Conwy and Camel estuaries during the first wave of the Covid-19 pandemic, probability of exceedance maps were produced for the Conwy and Camel model domains. 250 copies/l was taken as a minimum level of contamination required at the sampling source to be practically detectable and provide a RT-qPCR signal after concentration and extraction of the virus, based on the author’s field experience. The percentage of time at each model node where tracer concentrations exceeded this threshold were calculated using a Matlab script for the period 1st February–1st July 2020 and the results were interpolated between model nodes to produce coloured risk maps. The 250 copies/l detectability threshold is also identified with a dashed grey line on each time-series plot.

## 3 Results and discussion

### 3.1 Hydrology

**Figure 5:**
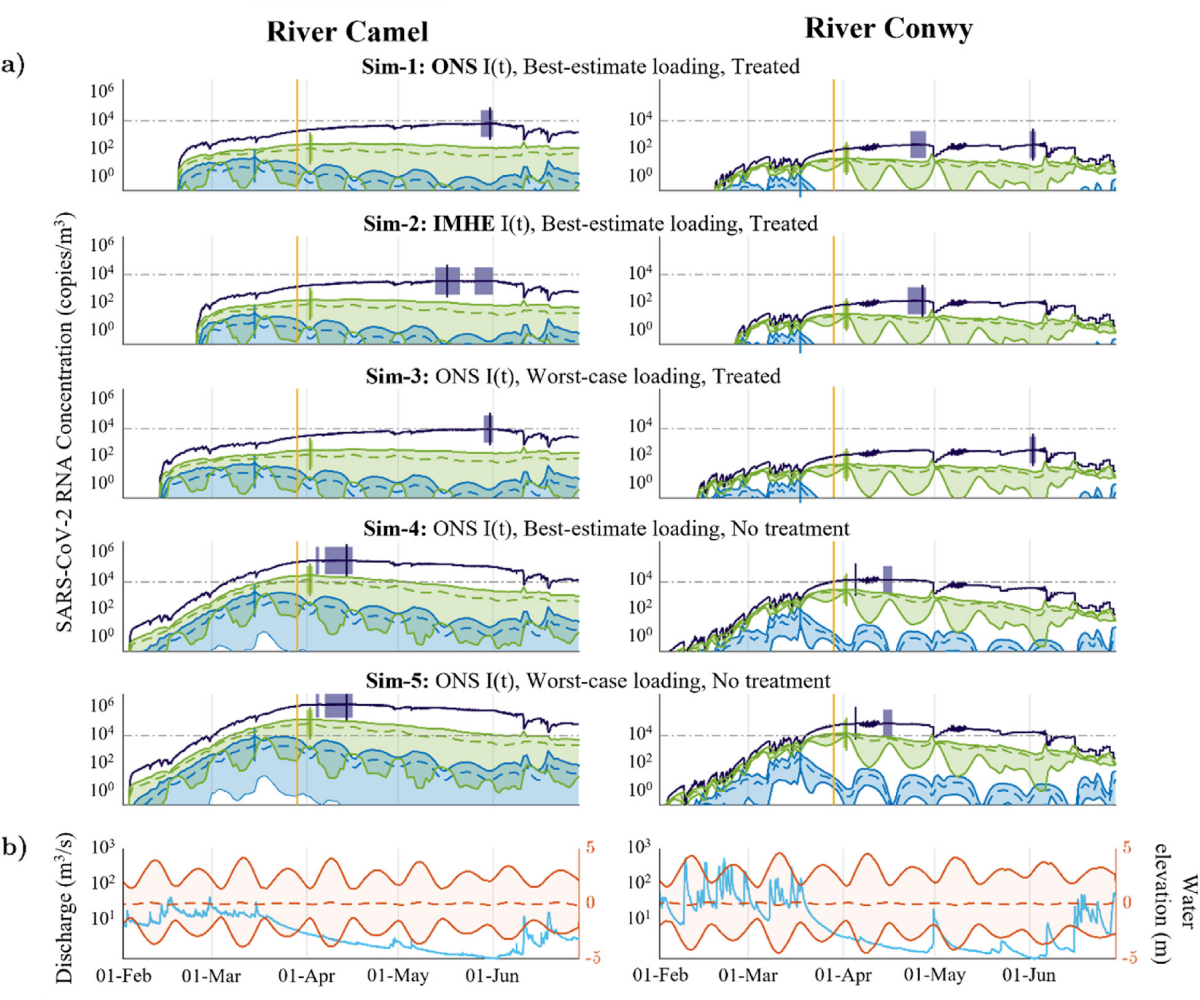
(a) Timeseries of modelled SARS-CoV-2 RNA concentrations where T_90_ = 24 h at three locations in the Camel/Conwy rivers and estuaries; 50m (purple) and 7.5km (green) downstream of the largest loading source in each river, and at the estuary mouth (blue). Mean daily averages (dashed lines) and mean daily max and minimum values (solid lines enclosed by colour shading) are presented for locations where significant diurnal variations occur due to the tide (blue and green timeseries). For each timeseries the date of the maximum daily SARS-CoV-2 RNA concentration is marked with a vertical coloured line and any dates where the concentration is greater than 95% of the max mean daily are highlighted with a colour block. Yellow vertical lines highlight the date of the peak of infections (29th March). Grey dash-dotted lines show what is assumed to be the minimum practicable detectable contraction (250 copies/l). See Table 2 for modelling details of the Simulations 1–5. (b) Timeseries of discharge for each river (blue) and simulation surface elevation at the estuary mouth relative to the tidally average value for the study period (red). The dashed red line shows the mean daily surface elevation and the solid red bounding lines plot the daily max and minimum surface elevations.

The Spring of 2020 was relatively dry in both the Camel and Conwy catchments resulting in declining base-flow of hydrographs from late February though to early June (Figure 5b). Mean discharge during February was 14.5 m^3^/s (Camel) and 83.3 m^3^/s (Conwy), whilst during May the mean discharge had declined in both rivers to 1.25 m^3^/s and 2.26 m^3^/s, respectively. Large discharge events (Q > Q_95_) predominantly occurred from February to mid-March, prior to the peak of infections (which occurred on 29th March) and again in June after the number of infections has subsided significantly. The only exception of an event above Q_95_ was in the Conwy on 30th April (Q_max_ = 21.8 m^3^/s) that occurred during neap tides – the impacts of this event on viral concentrations is discussed below. Two less significant discharge events occurred around the same period in the Camel on 29th April (Q_max_ = 3.1 m^3^/s) and 5th May (Q_max_ = 2.75 m^3^/s) and, being smaller, these events simulated negligible effects on within-estuary viral concentrations.

### 3.2 SARS-CoV-2 RNA: 1 day decay

Timeseries for Simulations 1–5 with *T*_*90*_ set to a minimum of 1 day are displayed in Figure 5a, which were extracted from the model domains at three locations within each estuary: (1) 50 m downstream; (2) 7.5 km downstream from the largest WWTP source; and (3) at the estuary mouth which is respectively 13.6 km and 21 km downstream from largest WWTP source in the Camel and Conwy. Simulations 1–5 represent ONS and IMHE best-estimate/worst-case infection data with and without wastewater treatment and inputted as a rising and then declining load, as shown in Figure 3b and Table 2. An interesting feature of the results from Simulations 1–3, where wastewater treatment was applied, is that the concentration of SARS-CoV-2 RNA in the river 50 m downstream of the tracer source continued to rise long after the peak of the infections (29th March; Figure 5a) – reaching maximums 1–2 months later: on the 31st May (Camel) and 2nd June (Conwy) in Simulations 1 and 3, and on the 17th May (Camel) and 27th Apr (Conwy) in Simulation 2. There are three main factors which contributed to these lags and the sustained elevated concentrations of SARS-CoV-2 RNA in the estuaries into the summer:

(1) River discharge was generally declining over the course of the spring (Figure 5b) resulting in a decreasing dilution effect from river flow and increasing estuarine residence times.
(2) The concentrations of the tracer loadings in Simulations 1–3 were relatively insensitive to the change in infections (e.g., a reduction of ∼5×10^4^ copies/m^3^/day: Figure 3) relative to the simulated river dilution effect (e.g., ∼2 × 10^5^ m^3^/day: Figure 5).
(3) There is a small cumulative concentration effect: Although 90% of the virus decays by the end of 1 day, 10% persists for longer.

In Simulations 4–5, where no wastewater treatment was applied, the second of the factors above did not apply and therefore the lag between the peak in Covid-19 infections and the peak concentration of SARS-CoV-2 RNA in the river/estuary was shorter, being 1–2 weeks: peak concentrations of viral RNA 50 m downstream occurred on the 14th April (Camel: 1.75 × 10^6^ copies/m^3^) and 5th April (Conwy: 1.2×10^5^ copies/m^3^).

Further downstream, 7.5 km from the largest WWTP source, the lag between the peak of infections and peak concentrations of SARS-CoV-2 RNA was shorter, with the peak occurring four days later (2nd April) in Simulations 1–5. Even further downstream, at the estuary mouths, the peak of concentrations actually proceeded the peak of infections occurring in Simulations 1–5 on 15th March (Camel) and 18th March (Conwy). Two factors that drove this shortening and eventually reversing of lag times were:

(1) The decreasing relative influence of river discharge on effluent dilution relative to dilution from the sea meant that the effect of the decline in river base-flow on dilution diminished.
(2) When the river discharge was greater (mainly in the spring) the faster streamflow advected the tracer downstream more rapidly allowing greater concentrations to reach the estuary mouths before significant decay had occurred (Figure 4).

Further evidence of the rapid flushing and transport effect of high discharge on SARS-CoV-2 RNA concentrations can be seen in Figure 5a during high flow events in late spring when the base-flows were relatively low, for example in the Camel estuary on 11th and 19th June and in the Conwy estuary on 30th April and 6th June. Large and flashy river discharge events on these dates resulted in a sharp drop in SARS-CoV-2 RNA concentrations close to the WWTP sources, generally by an order of magnitude but depending on the modelled scenario, and an increase in concentrations further downstream. Although SARS-CoV-2 RNA concentrations were largely restored to values simulated pre-discharge event, after a few days in the lower/mid estuaries and up to one week in the upper estuaries, which is consistent with previous studies (Turrell et al., 1996).

**Figure 6:**
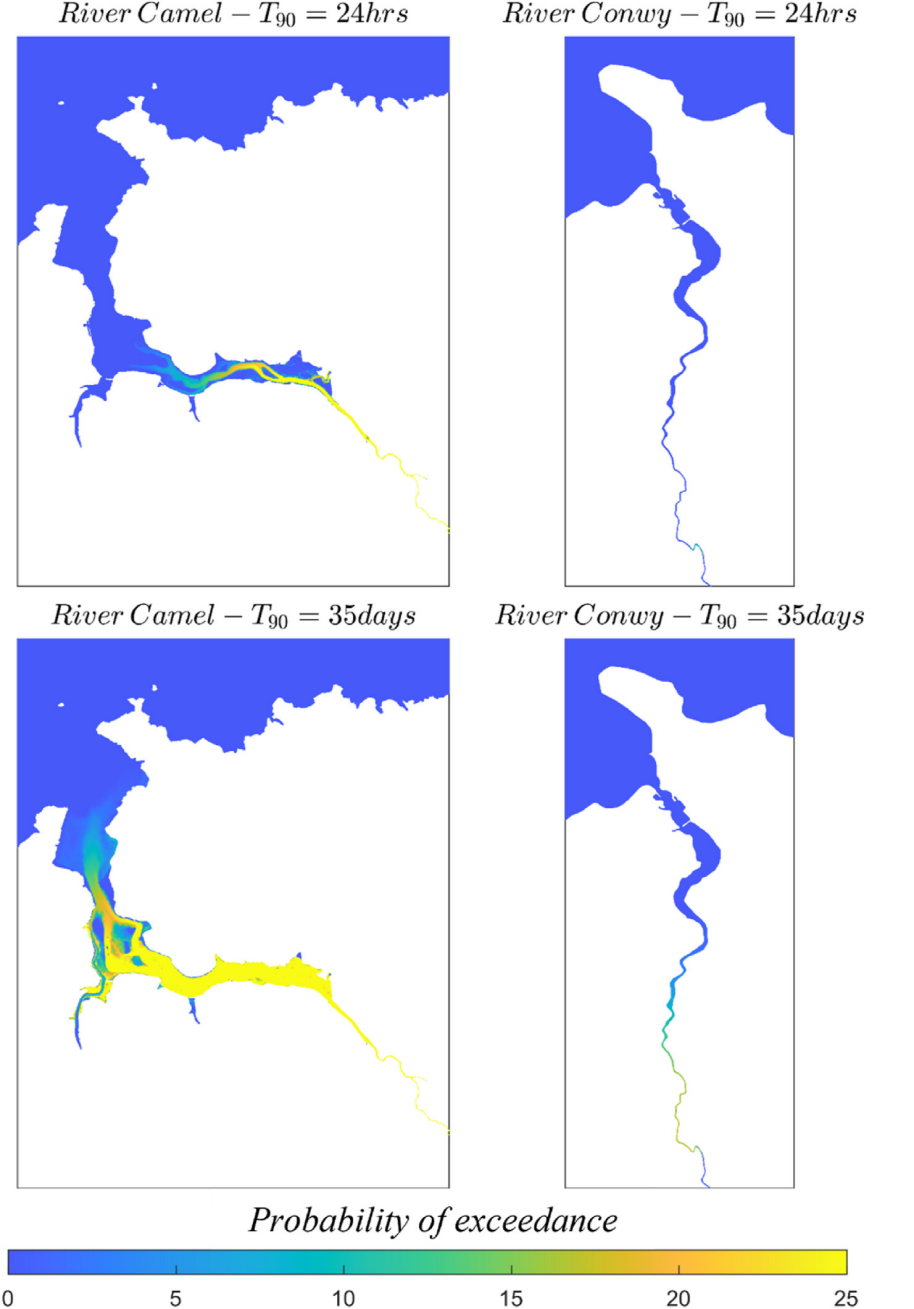
Probability of exceedance; SARS-CoV-2 RNA Concentration > 1 × 10^4^ (copies/m^3^) between 01/02/2020 and 01/07/2020. All panels show results from Simulation 4 (ONS best-estimate loading with no treatment; see Table 2 for further details).

Model outputs from Simulations 1–5 indicate that SARS-CoV-2 RNA concentrations were significantly lower in the Conwy than Camel, due to the lower population (and therefore loading) and generally higher river discharge causing greater dilution. Also, the Camel estuary mouth contained higher SARS-CoV-2 RNA concentrations than the Conwy mouth, presumably because the Camel is almost half the length of the Conwy. In the Camel estuary, Simulations 1–3 suggest that SARS-CoV-2 RNA concentrations fell below the detectability threshold of 250 copies/l within 50 m of WWTP output. Only in Simulations 4–5, where no treatment was assumed, did concentrations in the rivers/estuaries exceed the detectability threshold. As is evident in the Conwy in both Figures 5 and 6, in the case of Simulation 4, which was the untreated best-estimate scenario, even when very close to the main WWTP input the concentrate of SARS-CoV-2 RNA only exceeded the detectability threshold very briefly in the river. In the Camel, the detectability threshold was exceeded for a much longer period and spatially more extensively (beyond 7.5 km downstream of the largest WWTP input).

### 3.3 SARS-CoV2 RNA: 35 days decay

Increasing the decay time from 24 h to 35 days (840 h) had negligible impact on SARS-CoV-2 RNA concentration timeseries close to sources (Figure 8). However, further downstream, maximum concentrations increased, and higher concentrations were sustained for longer in all Simulations 1–5. For example, in Simulation 1, the Camel maximum concentrations 7.5 km downstream increased from 568 to 1786 copies/m^3^ and from 12 to 192 copies/m^3^ at the estuary mouth.

**Figure 7:**
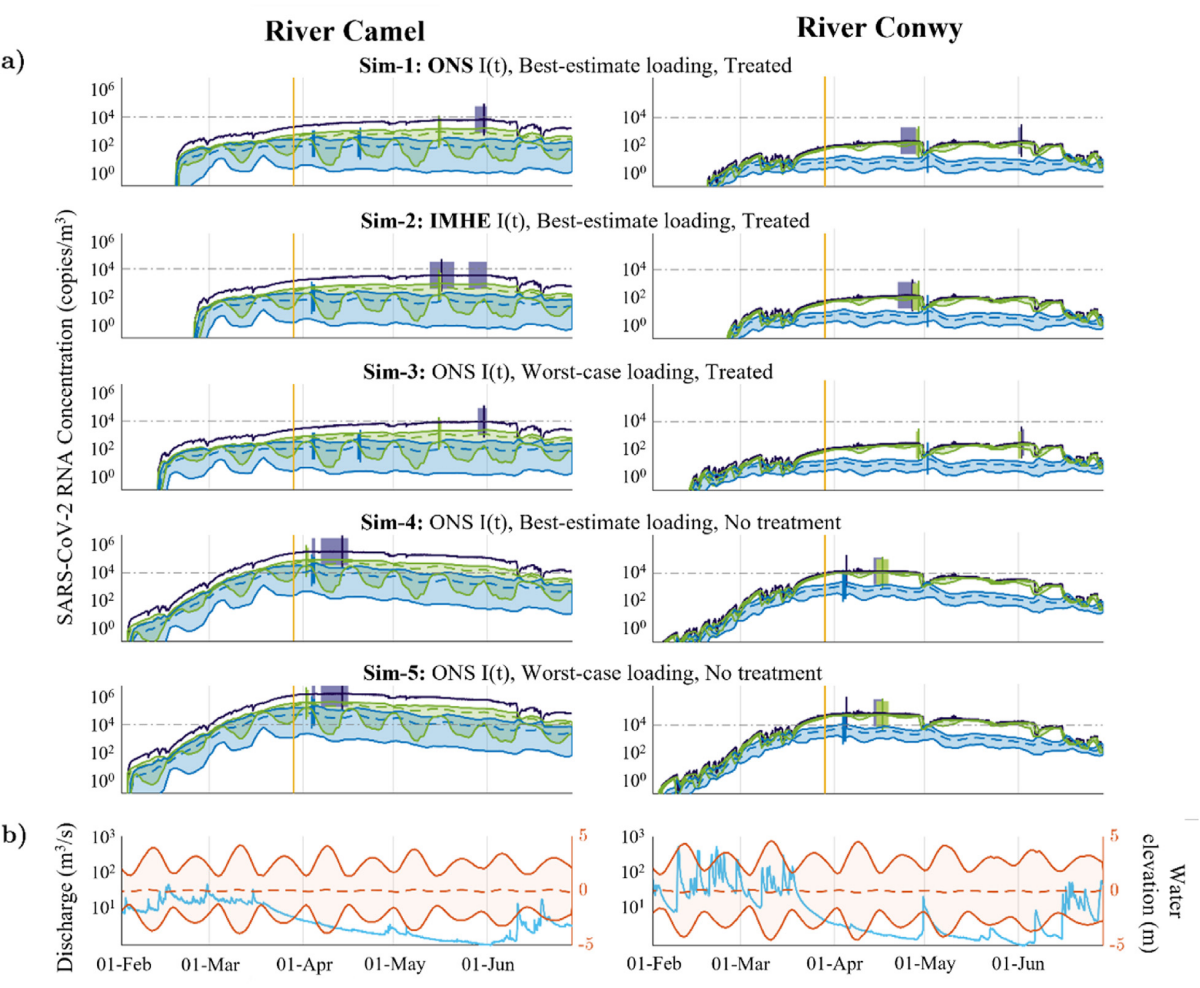
(a) Timeseries of modelled SARS-CoV-2 RNA concentrations where T_90_ = 35 days at three locations in the Camel/Conwy rivers and estuaries; 50m (purple) and 7.5km (green) downstream of the largest loading source in each river, and at the estuary mouth (blue). Mean daily averages (dashed lines) and mean daily max and minimum values (solid lines enclosed by colour shading) are presented for locations where significant diurnal variations occur due to the tide (blue and green timeseries). For each timeseries the date of the maximum daily SARS-CoV-2 RNA concentration is marked with a vertical coloured line and any dates where the concentration is greater than 95% of the max mean daily are highlighted with a colour block. Yellow vertical lines highlight the date of the peak of infections (29th March). Grey dash-dotted lines show what is assumed to be the minimum practicable detectable contraction (250 copies/l). See Table 2 for modelling details of the simulations 1–5. (b) Timeseries of discharge for each river (blue) and simulation surface elevation at the estuary mouth relative to the tidally average value for the study period (red). The dashed red line shows the mean daily surface elevation and the solid red bounding lines plot the daily max and minimum surface elevations.

An interesting effect of increasing the decay rate from 1 to 35 days was that concentrations 7.5 km downstream in the Conwy became less sensitive to tidal fluctuations; this can be seen by contrasting the green timeseries in the Conwy between Figures 5 and 7. When *T*_*90*_ = 1 day, the concentration of SARS-CoV-2 RNA was markedly diluted by tidal fluxes, except during neap tides when the tidal flux was reduced. However, when *T*_*90*_ = 35 days, concentrations of SARS-CoV-2 RNA were less sensitive to spring/neap tidal fluxes and followed a similar pattern 7.5 km and 50 m downstream, except during particularly large spring tides (for example on 9th April) when a small amount of tidal dilution was evident. Behind this change in behaviour at the 7.5 km downstream point was that, with decay set at *T*_*90*_ = 35 days, SARS-CoV-2 RNA was able to proliferate further downstream before decaying (recall from Figure 4 that it can take several days for terrestrial water to reach the estuary mouth) and therefore water pushed upstream by the incoming tide had a smaller dilution effect as it was contaminated with residual SARS-CoV-2 RNA. In contrast, in the Camel the tidal flux was still a clear driver of concentrations at the 7.5 km downstream point when *T*_*90*_ = 35 days, which can be attributed the fact that this point is closer to the estuary mouth in the Camel (Figure 1) than the Conwy and therefore more influenced by the tide.

**Figure 8:**
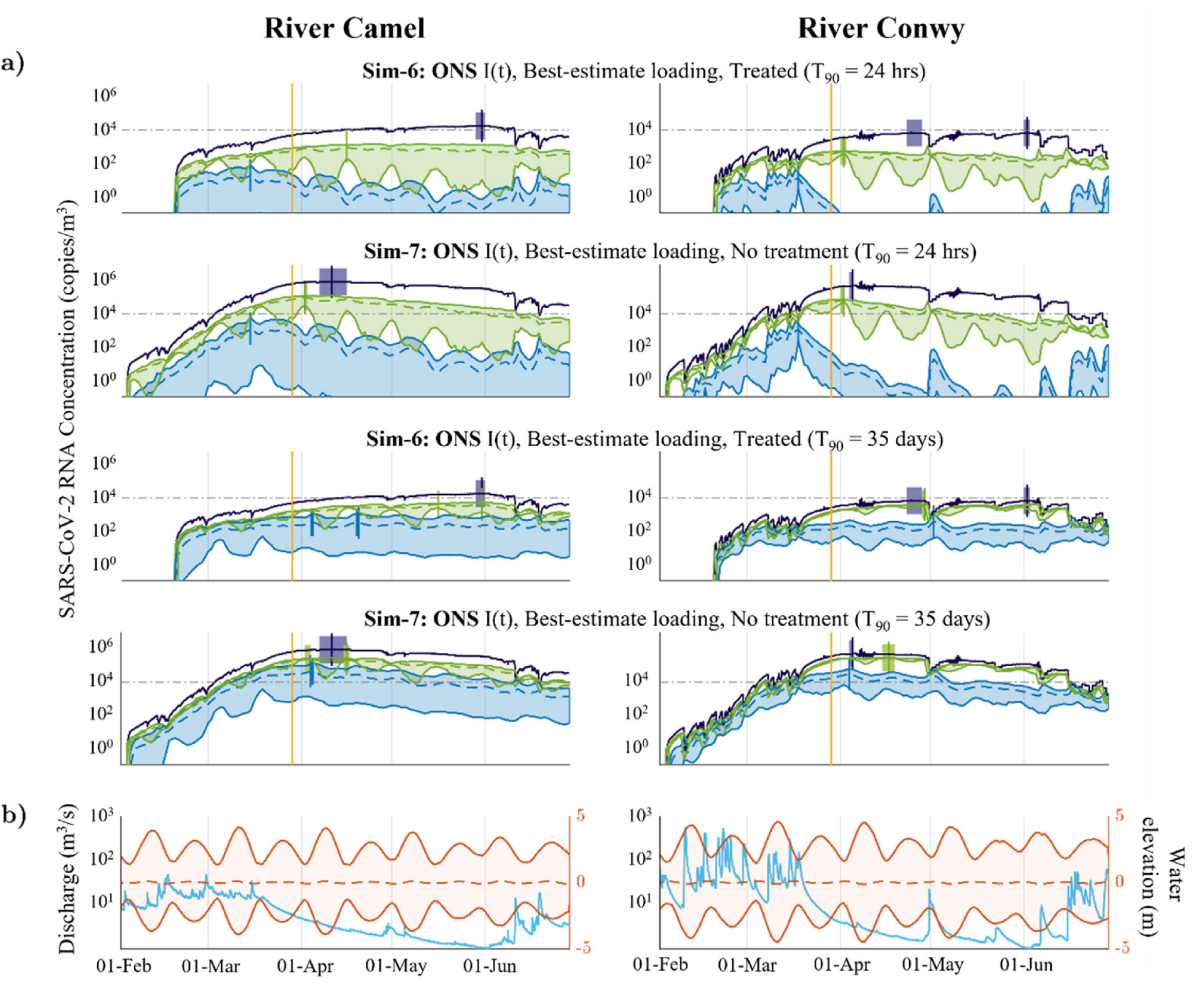
(a) Timeseries of modelled SARS-CoV-2 RNA concentrations in the Camel/Conwy rivers and estuaries where each river is subject to the same loading of SARS-CoV-2 RNA for T_90_ = 24 h and T_90_ = 35 days. Timeseries are plotted 50m (purple) and 7.5km (green) downstream of the largest loading source in each river, and at the estuary mouth (blue). For clarity of presentation mean daily averages (dashed lines) and mean daily max and minimum values (solid lines enclosed by colour shading) are presented for locations where significant diurnal variations occur due to the tide (blue and green timeseries). For each timeseries the date of the maximum daily SARS-CoV-2 RNA concentration is marked with a vertical coloured line and any dates where the concentration is greater than 95% of the max mean daily are highlighted with a colour block. Yellow vertical lines highlight the date of the peak of infections (29th March). Grey dash-dotted lines show what is assumed to be the minimum practicable detectable contraction (250 copies/l). See Table 2 for modelling details of the Simulations 6–7. (b) Timeseries of discharge for each river (blue) and simulation surface elevation at the estuary mouth relative to the tidally average value for the study period (red). The dashed red line shows the mean daily surface elevation.

In common with the *T*_*90*_ = 1-day Simulations 1–3, when *T*_*90*_ = 35 days the detectability threshold of 250 copies/l was not exceed at any point 50 m or more downstream of the largest source in either river. However, in the untreated Simulations 4–5 the detectability threshold was exceeded much further downstream and for a greater length of time in both rivers (Figures 5 and 6). In the Camel, Figure 7 shows that this detectably threshold was exceeded down into the middle of the estuary for over 25% the period 1st Feb through to 1st July for Simulation 4. However, in the Conwy, detectable concentrations did not reach the estuary in Simulation 4 (Figure 7) and even under the worst-case scenario conditions of Simulation 5 the detectability threshold was never reached at the estuary mouth.

### 3.4 Comparison of catchments with equal loading

SARS-CoV-2 RNA concentrations followed broadly similar spatial and temporal trends in Simulations 6 and 7 where it was assumed that tracer loading to both catchments was equal and from a single source (see Table 2 for further details of simulations). Three key differences were, however, evident (Figure 8). Firstly, concentrations of SARS-CoV-2 RNA were generally lower in the Conwy than the Camel due to higher river discharge and therefore greater dilution. Secondly, RNA concentrations were diluted more frequently and to a higher extent in the Conwy, due to more flashy discharge events in the Conwy. Thirdly, there were differences between the dates on which maximum RNA concentrations occurred or where concentrations were within 5% of maximum – generally later in the Conwy than Camel, due to the differences in estuary lengths and hence dispersion travel times.

### 3.5 Implications

Whilst the simulations described above have been parameterised based on best-estimate and worst-case SARS-CoV-2 infections in two UK catchments during the first wave of the Covid-19 pandemic, our methodology is of relevance to other viral pathogens that pose an exposure hazard to humans. Further, there is still scope for evidence to emerge that SARS-CoV-2 is infectious in very low doses, and that the model applies to aquatic wildlife that could also be infected by SARS-CoV-2 and act as a reservoir, although this relies on the virus remaining intact.

Although there is conflicting evidence for viable SARS-CoV-2 particles remaining infectious in human stool (Guo et al., 2021; Pedersen et al., 2021), it is unlikely that the virus remains intact following transfer through the WWTP and subsequent discharge into the environment. However, since the beginning of the COVID-19 pandemic there has been speculation about the role of sewage and surface waters for environmental transmission of SARS-CoV-2 (Jones et al., 2020). Infectious virus particles discharged into the environment could pose a risk for humans who come in contact with receiving waters, and there is even a theoretical risk of zooanthroponotic spillover into animals (Mathavarajah et al., 2021a, 2021b). Under experimental conditions, infectious SARS-CoV-2 particles can remain stable in river water for several days, particularly at lower temperatures (Sala-Comorera et al., 2021). Yet, despite much supposition there remains no evidence for the presence of infectious, replication-capable, SARS-CoV-2 particles in environmental faecal wastes or waters (Sobsey, 2022). Coronaviruses are characterised by a lipid envelope, which becomes compromised by the detergents and solvents inherent in wastewater, making infectious virus particles no longer viable (Gundy et al., 2009). The half-life of SARS-CoV-2 RNA in wastewater depends on environmental conditions (e.g., temperature; Yang et al., 2022), but detecting the signal from these remaining fragments of RNA provides a powerful tool for the monitoring and surveillance of upstream levels of community infection (Ahmed et al., 2020a, 202b).

Our scenario-based modelling has shown the potential, case-specific behavioural dynamics of SARS-CoV-2 RNA following discharge into rivers and into the coastal zone, which could have important implications for future monitoring and surveillance strategies. Although our models are informed by the quantification of SARS-CoV-2 RNA, our approach could also be applicable to the behaviour of other human viruses discharged from WWTPs into receiving surface waters. SARS-CoV-2 RNA isolated from the environment has been extracted from either intact or partially lysed virus particles, where the nucleic acid will still be associated with remnants of the envelope and viral capsid. It is less likely that significant concentrations of isolated RNA are present in surface waters due to the half-life of naked RNA in the environment. Therefore, the behavioural dynamics and transport modelled in the two rivers in this study could be transferable to the dynamics of other enveloped viruses. Non-enveloped RNA viruses, such as norovirus and rotavirus, which are two of the most common causes of viral gastroenteritis, are also commonly discharged from WWTPs into receiving waters (Cuevas-Ferrando et al., 2022). Such enteric viruses can remain stable in the environment for extended periods and pose public health risk to those exposed to contaminated water (Dean and Mitchell, 2022) – in which case model insights from the *T*_*90*_ = 35 days simulations are particularly pertinent. The comparative behaviour of enveloped and non-enveloped viruses in the water column remains unclear, but our scenario-based modelling provides a first indication of how virus particles can move through different environmental matrices within contrasting catchments, from the point of discharge through transitionary waters and out into the coast.

### 3.6 Model limitations

Both the Conwy and Camel estuaries, like many estuaries in Britain, are predominantly partially or well-mixed vertically, due to shallow water depths (generally <10 m) and strong macro tidal mixing, even though freshwater inputs can be large. Depth-averaged models have been shown to reproduce the correct along-channel salinity gradients in the Conwy (Robins et al., 2014), and are therefore considered appropriate for this study, provided that the parameterisations of bathymetry and discharge are appropriately resolved (e.g., sub-daily discharge forcing: Robins et al., 2018). Nevertheless, these systems do experience three-dimensional flows associated with estuarine fronts and stratification (Howlett et al., 2015), axial convergent fronts (Brown et al., 1991), tide-induced eddies (Geyer and Signell, 1992), wind-driven flows and wave-current interaction (Wolf and Prandle, 1999) – and future studies that incorporate these processes could add a higher grain of detail to the simulations; again, provided that the model spatio-temporal resolution is not compromised. Finally, our models do not consider the behaviour of SARS-CoV-2 RNA when it becomes associated with particulate matter, or other contaminants, in wastewater or surface waters. Recently, it has been demonstrated that both enveloped and non-enveloped viruses are able to bind to microplastics in surface water and remain infectious for a number of days (Moresco et al., 2022). It has also been proposed that SARS-CoV-2 and other respiratory viruses could theoretically bind to microplastics, which could facilitate increase dissemination and transport within the environment (Zhang et al., 2022; Moresco et al., 2021).

## 4 Conclusion

The initial aim of this study was to assess whether SARS-CoV-2 RNA was likely to have been present in detectable quantities in UK rivers and estuaries during the first wave of the Covid-19 pandemic. Through estuary modelling with realistic viral loading that was parameterised on the Camel and Conwy catchments (UK), we show that this was likely in some but not all scenarios, being contingent primarily on wastewater treatment, hydrology, and downstream location.

Secondly, our aim was to assess the sensitivity of SARS-CoV-2 RNA concentrations in these systems to influent concentrations, WWTP treatment, viral decay rates and catchment hydrology, tidal process and estuary shape. The survival of detectable viral RNA from a catchment will be sensitive to the gradation of the catchment which translates into the flow of the river. The faster the river, the faster the intact virus can reach bathing waters and shellfish beds. The abundance of WWTPs and CSOs discharging to rivers with higher flows is an important factor in assessing the hazard posed by microorganisms within wastewater to human health and food security. Dilution can be an important factor, but due to the high concentration of microbial analytes, the level of dilution is infrequently sufficient to dilute the analytes to levels considered ‘below the limit of detection.’ There is an increased hazard posed by analytes with a *T*_*90*_ > 24 h, owing to their potential to concentrate in the brackish freshwater-saltwater interface due to tidal effects. This phenomenon can be important for risk assessing bathing water and shellfish bed safety.

## Declarations

### Author contribution statement

Peter Robins & Neil Dickson: Conceived and designed the experiments; Performed the experiments; Analysed and interpreted the data; Contributed reagents, materials, analysis tools or data; Wrote the paper.

Shelagh Malham & Davey Jones: Conceived and designed the experiments; Analysed and interpreted the data; Contributed reagents, materials, analysis tools or data; Wrote the paper.

Jess Kevill; Andrew Singer & Richard Quilliam: Analysed and interpreted the data; Wrote the paper.

### Funding statement

The authors were supported by 10.13039/501100000270Natural Environment Research Council [NE/S004548/1, NE/S005196/1, NE/V010441/1].

### Data availability statement

Data will be made available on request.

### Declaration of interest’s statement

The authors declare no conflict of interest.

### Additional information

No additional information is available for this paper.

## Figures and Tables

**Table:** 

Ref	Site Name	Treatment	Effluent loading weighting	Location of Discharge in Model
**Camel Catchment**				
Cam01	Bilsland STW	Biological (2°)	0%∗	Excluded
Cam02	Bodmin Nanstallon STW	UV (3°)	0.2%	Redistributed
Cam03	Bodmin Nanstallon STW	UV (3°)	0%∗	Excluded
Cam04	Bodmin Scarletts Well STW	UV (3°)	0.1%	Redistributed
Cam05	Camelford Station STW	Biological (2°)	0%∗	Excluded
Cam06	Camelford STW	Chemical (2°)	24.2%	River Camel
Cam07	Chapel Amble	Readbed (2°)	1.4%	River Amble
Cam08	Delabole STW	Chemical (2°)	17.1%	River Allen
Cam09	Dwellings at Wadebridge STW	Package (2°)	0.3%	Redistributed
Cam10	Hawkers Cove STW	Biological (2°)	0.6%	Redistributed
Cam11	Helstone STW	Biological (2°)	0%∗	Excluded
Cam12	Little Petherick STW	UV (3°)	0.0%	Redistributed
Cam13	Porthilley STW	UV (3°)	0.1%	Redistributed
Cam14	St Breward STW	Biological (2°)	22.1%	River Camel
Cam15	St Mabyn STW	Biological (2°)	20.1%	River Allen
Cam16	St Teath STW	Biological (2°)	13.6%	River Allen
Cam17	Wadebridge STW	UV (3°)	0.2%	Redistributed
**Conwy Catchment**				
Con01	Betws-y-Coed WWTW	Biological (2°)	10.4%	River Conwy
Con02	Capel Curig SD Scheme	Biological (2°)	0.0%	River Conwy
Con03	Capel Garmon STW	Biological (2°)	0.0%	River Conwy
Con04	Craig Dinas STW	Biological (2°)	0.1%	River Conwy
Con05	Cwm Penmachno STW	Biological (2°)	1.1%	River Conwy
Con06	Dolgarrog/Tal-y-Bont STW	Biological (2°)	8.4%	River Conwy
Con07	Dolwyd STW	Septic Tank (1°)	0%∗	Excluded
Con08	Dolwyddelan WWTW	Biological (2°)	4.3%	River Conwy
Con09	Eglwysbach STW	Biological (2°)	4.4%	River Conwy
Con10	Glasfryn STW	Biological (2°)	0.2%	River Conwy
Con11	Graig STW	Package (2°)	0.2%	River Conwy
Con12	Groesffordd STW	Biological (2°)	0%∗	Excluded
Con13	Herhyd	Biological (2°)	0.8%	River Conwy
Con14	Llan Penmachno STW	Biological (2°)	3.1%	River Conwy
Con15	Llanwrst STW	Biological (2°)	16.8%	River Conwy
Con16	Mountain View	Biological (2°)	0.0%	Excluded
Con17	Nebo	Biological (2°)	0.3%	River Conwy
Con18	Penmaenmawr WWTW	Biological (2°)	41.1%	Conwy Bay Coast
Con19	Pentrefelin STW	Biological (2°)	0.3%	River Conwy
Con20	Pentrefoelas STW	Biological (2°)	0.7%	River Conwy
Con21	Rowen STW	Biological (2°)	1.7%	River Conwy
Con22	Trefiw STW	Biological (2°)	4.1%	River Conwy
Con23	Tyn Y Groes STW	Biological (2°)	1.1%	River Conwy
Con24	Ysbyty Ifan	Biological (2°)	0.8%	River Conwy

## Appendix 1 Apportioning of effluent load to WWTPS for the Camel and Conwy catchments. ∗Estimates were not recorded for some small scale WWTP by Cefas (2014, 2015); these were excluded from the models.

National River Flow Archive, 2020a, National River Flow Archive, 2020b, van Kampen et al., 2021, Wu et al., 2020
